# An Overview of Our Current Understanding of Diabetic Macular Ischemia (DMI)

**DOI:** 10.7759/cureus.3064

**Published:** 2018-07-30

**Authors:** Muhammad Usman

**Affiliations:** 1 Internal Medicine, Jinnah Hospital Lahore (JHL)/Allama Iqbal Medical College (AIMC), Lahore, PAK

**Keywords:** dmi, diabetic macular ischemia, diabetes, retinopathy

## Abstract

Diabetic macular ischemia (DMI) is a troublesome complication of diabetes mellitus. The pathogenesis, progression, consequences, and treatment options for this disease are still poorly understood. However, it is believed that this complication is associated with several risk factors like poor glycemic control and high blood pressure. Two factors have been identified in the pathogenesis of the disease that play pivotal roles in disease development and progression. One of these factors includes extensive damage to the microvasculature of the retina. This includes narrowing of the vascular lumen and extensive damage to the endothelial cells, pericytes and the extracellular matrix. The second factor includes extensive damage to the neurosensory layer of the retina. DMI is always associated with other complications of diabetic retinopathy like retinal edema, so it is very difficult to find an isolated case of DMI. Moreover, the condition is also very rare making it very difficult to study and diagnose. However, a number of diagnostic tests like optical coherence tomography (OCT), fluorescence angiography (FA), and perimetry can be used as tools for the early detection of DMI. Since this disease damages the retina, loss of vision is an inevitable consequence that can progressively worsen over time. Also, DMI has been implicated as a risk factor for retinal edema and progressive diabetic retinopathy. Until now, no defined treatment protocol has been devised. The only available treatments focus on the management of risk factors (hyperglycemia and hypertension). Still, many aspects of DMI remain poorly studied and understood. This review paper aims to add to our current understanding of diabetic macular ischemia (DMI).

## Introduction and background

 As per 2013 statistics, diabetes has become a global burden to more than 382 million people. These numbers are expected to rise to 592 million by 2035 [1]. Diabetes is not merely an increase in the concentration of blood glucose; it also comes with a handful of other complications including nephropathy, neuropathy, and retinopathy [2]. Diabetic retinopathy is perhaps one of the most cumbersome complications of diabetes characterized by interrelated changes in the retina of the diabetic patient. Until now, much attention has been paid to retinal ischemia as it has been considered the primary risk factor for proliferative diabetic retinopathy. But little research has been done to describe ischemia of the macular region of the eye, mainly due to difficulty in its detection and limited treatment options. Extensive retinal capillary non-profusion has been reported in the cases of diabetic macular ischemia (DMI), as seen through ophthalmological examinations [3]. Histological examinations also reveal acellular capillaries in the area where the blood supply is compromised [4]. The progression and risk factors for diabetic retinopathy and DMI are perhaps the same. The progression of both diseases, perhaps, includes occlusion of capillaries in the early stages and the pre-capillary arterioles in the later stages of the diseases. Moreover, the risk factors for both diseases include hyperglycemia and hypertension [5-7]. In addition, DMI has been related to some other risk factors as well: age of onset of the disease, macular edema associated with diabetes, and advancing stages of diabetic retinopathy [8-9]. Although the pathogenesis, risk factors, and treatment options for DMI are poorly defined and studied, it still remains an important cause of vision loss, as seen in diabetic patients.

The purpose of this article is to elucidate all possible aspects of DMI in the light of the current literature.

## Review

Pathogenesis of DMI

Characteristically, two anatomical changes can be seen in the retina of patients with DMI. First, due to pronounced cellular and extracellular damage, there is an extensive loss of neuro-retinal tissues. Second, there is marked occlusion of the vessels supplying the retina [10]. These anatomical changes, however, occur in the later stages of the retinopathy along with a set of other complications like retinal edema. That is why it is quite difficult to anatomically characterize and observe macular diabetic ischemia in isolation. The anatomical and physiological basis of this disease is still very poorly studied. 

The pathological changes that most likely lead to the occlusion of capillaries can be divided into three categories: changes in the lumen itself, changes in the cellular components of the vessel wall (and supportive tissue such as the basement membrane), and alterations in the extracellular components. Diabetes happens to induce pathologies of all these mentioned structures. The earliest detectable pathology in DMI is a change in the structure of the retinal capillaries' basement membrane. Normally, the basement membrane has a thin, fine structure made of type IV collagen fibers. Its functions include giving support to the capillary endothelial cells and pericytes, acting as a sieve for filtering out structures, and putting a stop to proliferation. But in cases of DMI, the basement membrane shows an abnormally thick structure and becomes rich in type IV and type III collagen fibers. In addition, the ground substance of basement membrane shows a decreased quantity of heparin sulfate [11-12]. These alterations in the structure of basement membrane might be the result of improper retinal-cellular function. Conversely, improper basement membrane structure might be responsible for cellular dysfunction since an abnormal basement membrane triggers a number of pathological processes, including increased production of vascular endothelial growth factor (VEGF) [10].

Another important capillary pathology seen in DMI is characteristic changes in the structure of pericytes. Pericytes are the cells that surround the retinal capillary lumen and provide it with structural and functional support. Structurally, they control the vessel lumen and synthesize the basement membrane. Functionally, these cells control the division and differentiation of endothelial cells of retinal capillaries [13]. Pericytes are easily visible in enzymatic digest preparations. In these preparations, pericytes are visible in the form of round cells with prominent nuclei surrounding the capillary lumen. But in DMI, they undergo apoptosis and are seen as empty (balloon-like) spaces [10]. One of the major consequences of pericyte apoptosis is occlusion of the retinal capillary lumen. 

Another characteristic feature is the change in the endothelial cells following diabetic retinopathy. There is extensive damage to endothelial cells of capillaries in the DMI, and underlying causes are variable but still poorly understood. It is believed that changes during diabetes that lead to enhanced endothelial damage include an increase in the level of inflammatory cytokines, enhanced aggregation, and activation of the platelet and clotting cascade, and increased aggregation and decreased deformability of red and white blood cells [10, 14-15]. This damage to the endothelial cells ultimately leads to the occlusion of the retinal capillary lumen.

The damage to the neurosensory retina and the retinal blood vessels is an interconnected process, i.e. damage to the blood vessels of the retina would lead to retinal neurosensory damage and neurosensory damage would induce retinal capillary damage. For instance, the hypoxia and damage of retina caused by the occlusion of retinal blood vessels would trigger a protective response in the retina. As a response, the retina increases the production of VEGF. VEGF, in turn, leads to enhanced endothelial cell damage, abnormal endothelial cell proliferation, migration, and altered endothelial cell junctions' permeability. Also, the damage to retinal neurosensory layers significantly reduces the secretion of several cytokines by the retinal cells. This also adds to the vascular occlusion. Furthermore, certain chemicals called prostanoids (most probably produced by oxidative damage caused to the lipids) can also compromise the blood flow to retinal blood vessels. Moreover, altered activation of N-methyl-D-aspartate (NMDA) and somatostatin receptors seem to play some role [10, 16-18]. These, and a number of other factors, add to the damage caused to the retina by diabetes.

Progression of DMI

The natural history of a disease describes the steps of progression, beginning from the start of the disease to its more advancing stages. The natural history of DMI is poorly understood. There are several reasons as to why the natural history of DMI is not clearly explained. One reason, as mentioned before, is the lack of identification of the disease. The second reason leading to the poor understanding of the clinical course of this disease is its rarity [10]. However, some attempts have been made where researchers have tried to explain how DMI progresses, but the studies have not been very effective. More work is needed in this area. The result of research data has shown that the severity of DMI does not aggravate much with time among its sufferers [19]. However, other studies have produced different results. It is now believed that the severity of the disease increases with time [20-21]. The ability of DMI to improve with time is not known. In fact, to date, there is no clinical trial or study that could analyze the potential of DMI to heal on its own. However, some studies do suggest that the loss of vision that usually follows DMI may get better with time. If this were to happen, then revascularization in the marginal zone of the degenerated macula might be the cause [22].

Clinical evaluation of DMI

As mentioned before, it is very rare to find an isolated case of DMI, and methods of clinical examination are not very definitive. But a number of diagnostic methods do exist that can be clinically used for the diagnosis of DMI. One such method is fundoscopy and fundus photography. One characteristic feature of DMI that can be appreciated upon fundoscopy, and fundus photography, is the presence of featureless areas of the ischemic retina where no exudates or blot hemorrhages are present [10]. Surrounding the area of ischemia is the area of hypoxia where dilated blood vessels can be found. In addition, larger caliber blood vessels that transverse the area of ischemia are highly attenuated and appear as ghost vessels. The macula is markedly depressed; this is due to the infarction of that area [10].

Another method that can be used in the evaluation and clinical diagnosis of DMI is fluorescein angiography (FA). In fact, this is the gold standard procedure that is used for the diagnosis of ischemic macular diseases. FA shows hyperfluorescent areas of the retina. In these areas, there is an absence of blood in the macular blood vessels. Surrounding the region of ischemia is the area with characteristic microvasculature distortion including arteriolar and capillary dilatation. Two basic findings are highly diagnostic for DMI. First, the foveolar avascular zone (FAZ) is widened and highly irregular [23-25]. Normally, the FAZ has a diameter between 0.12-1.20 mm. Normally, the mean vertical diameter is around 0.579 ± 0.015 mm [26]. In cases of DMI, the average diameter of FAZ can increase to as much as 0.94 mm. Second, the pattern of macular capillaries becomes highly irregular and spaced, indicating that there is some degree of intervening capillary loss. Surrounding the hypo-fluorescent areas are regions of hyperfluorescent leakage indicating the dilatation and attrition of surrounding arterioles and capillaries [27].

Optic coherence tomography (OCT) is another useful clinical tool that can be used for the detection of DMI. This method is based on the principle of detection of macular thickness. This is perhaps the most accurate and reliable way to determine macular thickness in retinopathies. In the early stages of diabetic retinopathy, the pericentral regions of the retina become thin. This thinning is perhaps due to the loss of neurosensory layers of the retina in the pericentral locations. Also, there is a significant decrease in the thickening of the nerve fiber layer of the retina. The ischemic areas of macula appear thin, in the absence of retina, during OCT investigation. However, the presence of edema and the presence of any coexisting retinal pathology- very common in diabetic retinopathy- usually make the results of OCT unreliable and difficult to interpret [23, 27-29].

All methods mentioned until now are useful in the detection of structural changes that occur during DMI. Perimetry, however, is a quick yet useful clinical investigation tool that can be used to detect the functional loss of vision that follows DMI. The results of perimetry typically show significant loss of vision. The results of perimetry correlate with the results of angiography. These results do confirm that areas of angiographic dropout show a decrease in visual acuity [10].

Another useful test that can be used to detect DMI is an electroretinogram (ERG). This method is based on evaluating the function of the diabetic retina. Therefore, this method can detect functional changes long before any structural changes can be observed on FA or OCT. The most consistent ERG finding in patients with DMI is an increase in the oscillatory potential implicit (OPI) time. This change suggests that there is some underlying pathology in the cells of the retina (bipolar, ganglion, and amacrine cells) [30].

In addition, some other diagnostic tests can also be performed. These tests include color perception testing and contrast sensitivity testing. Both of these tests show a progressive loss of color vision and contrast sensitivity of the eye, respectively, as pathologies progress [10].

Clinical significance of DMI

As yet, there is no defined treatment for DMI. However, a number of complications from DMI do exist, making this disease quite bothersome. A decrease in visual acuity is perhaps the most important consequence of DMI. Two factors are particularly important in determining the loss of vision as seen in DMI: enlargement of the foveolar avascular zone and enlargement of the perifoveal intercapillary area [31]. Enlargement of these areas is consistently related to a poor visual acuity score. For instance, data suggests that a visual acuity score of 20/50 is consistently present in patients with DMI with a FAZ >/= 0.55 mm2 or a perifoveal intercapillary area >/= 14,000 mm2 [31].

DMI is a source of severe vision loss, as mentioned earlier. But this loss of vision does not seem to improve even after the associated complications of diabetic retinopathy (retinal edema, for instance) is corrected. The data in this regard is, however, conflicting [32]. 

It is not surprising to know that DMI is associated with a poor prognosis of diabetic retinopathy. In fact, some studies have linked DMI as a risk factor for complications like progressive diabetic retinopathy and retinal edema [10]. For instance, the one-year risk of patients with DMI to develop progressive diabetic retinopathy was found to be almost 42%, which is significantly higher than the individual without DMI (the chance of progressive diabetic retinopathy in such individuals was found to be almost 18%) [33]. 

Treatment options for DMI

Until now, there is no defined cure for DMI. The only plausible cure seems to be the management of the underlying causes and risk factors. These include control of blood sugar levels and optimum blood pressure control. In addition, other risk factors like anemia and nephropathy should also be controlled [34].

One surgical procedure that has shown significant promise in the treatment of DMI is laser photocoagulation. This technique is mainly focused on correcting the capillary damage as seen in patients with DMI. This method has shown significant promise as it not only slows the progression of the disease, but also significantly improves the loss of vision as seen in patients with DMI [34]. But this method is associated with a long list of complications. Moreover, anti-VEGF drugs were thought to produce beneficial results (remember that increased production of VEGF by the damaged vessel cells is one of the fundamental reasons for the progression of these diseases), but the results have been disappointing [34]. 

Use of steroid drugs like fluocinolone acetonide has been suggested. These drugs are helpful in the management of diabetic retinopathy as these drugs can significantly reduce retinal edema. But their role in the cure of diabetic macular ischemia is still poorly understood [34].

## Conclusions

DMI is a troublesome complication of diabetes mellitus; the pathogenesis, cause, progression, and treatment options are poorly understood and studied. Much research is needed to clarify the mysteries of DMI. The paucity of research and limited treatment options make treating this condition quite challenging. Therefore, more research is needed in order to add to our current understanding of this condition and to explore the appropriate treatment options for this cumbersome diabetic complication. 
